# Optimus Primed: Media Cultivation of Robot Mental Models and Social Judgments

**DOI:** 10.3389/frobt.2020.00062

**Published:** 2020-05-07

**Authors:** Jaime Banks

**Affiliations:** College of Media and Communication, Texas Tech University, Lubbock, TX, United States

**Keywords:** trust, sympathy, characters, mental models, priming, heuristics, mentalizing, exemplification

## Abstract

Media influence people's perceptions of reality broadly and of technology in particular. Robot villains and heroes—from Ultron to Wall-E—have been shown to serve a specific cultivation function, shaping people's perceptions of those embodied social technologies, especially when individuals do not have direct experience with them. To date, however, little is understood about the nature of the conceptions people hold for what robots are, how they work, and how they may function in society, as well as the media antecedents and relational effects of those cognitive structures. This study takes a step toward bridging that gap by exploring relationships among individuals' recall of robot characters from popular media, their mental models for actual robots, and social evaluations of an actual robot. Findings indicate that mental models consist of a small set of common and tightly linked components (beyond which there is a good deal of individual difference), but robot character recall and evaluation have little association with whether people hold any of those components. Instead, data are interpreted to suggest that cumulative sympathetic evaluations of robot media characters may form heuristics that are primed by and engaged in social evaluations of actual robots, while technical content in mental models is associated with a more utilitarian approach to actual robots.

## Introduction

Media influence people's perceptions of reality broadly (Gerbner and Gross, 1976) and of technology in particular (Nisbet et al., 2002). Robot villains and heroes—from Ultron to Wall-E—serve a specific cultivation function, shaping people's perceptions of those embodied social technologies (e.g., Mara et al., 2013), especially when individuals do not have direct experience with them. To date, however, little is understood about the nature of the conceptions people hold for what robots are, how they work, and how they may function in society—as well as the media antecedents and relational effects of those cognitive structures. This study takes a step toward bridging that gap by partially replicating and extending one of the few empirical works in this domain (Sundar et al., 2016) to explore the relationships among individuals' recall of robot characters from popular media, their mental models for robots, and social evaluations of an actual robot. Findings suggest that mental models rely on a small set of common and tightly linked components (beyond which there is a good deal of individual difference), but robot character recall and evaluation have little association with whether people hold any of those components. Instead, data are interpreted to suggest that cumulative sympathetic evaluations of robot media characters may form heuristics that are primed by and engaged in social evaluations of actual robots, while technical content in mental models is associated with a more utilitarian approach to actual robots.

## Review of Literature

The prevalence of robot characters in popular media invites questions about the role that exposure to and evaluation of those characters may play in fostering people's understanding of robots, as well as fostering attitudes toward them and varied intentions to engage them. These potentials are perhaps best considered through the lens of media's influence on mental models for agents and interactions, as well as how those models may contribute to our understandings of social encounters.

### Mental Models as Ways of Knowing Robots

Mental models (MMs) are dynamic cognitive frameworks representing spatial, systemic, causal, or situational phenomena: structures of symbolic “tokens” that signify perceptible or abstract entities. Those relational structures correspond to given situations that they collectively represent (Rickheit and Sichelschmidt, 1999), such that MMs serve as interpretive frames for immediate experience (Craik, 1943). In other words, mental models are sets of ideas for what the world is, how it works, how it unfolds, or what happens in it, and these ideas are used to describe, explain, and predict events or things in the world. Just as one may use MMs of the actual world to guide engagement of fictional worlds (e.g., Krakowiak and Oliver, 2012; McGloin et al., 2015), MMs derived in whole or part from media experiences may be used to guide engagement of actual situations or agents. Understanding MMs' antecedents, formation processes, characters, and effects requires consideration of both MM contents (what are the cognitive frameworks' constitutive tokens?) and MM structure (how are tokens related to one another?).

The question of MM *content* is a matter of the knowledge held in one's internal representations of a phenomenon and the degree to which that knowledge corresponds with the actual phenomenon. MMs may contain tokens representing abstract or concrete elements and those representations may be objectively true, actually false, or constitute variably reliable heuristics (Byrne, 2005). The content of MMs is gleaned over time through personal and/or mediated experience (Seel and Strittmatter, 1989); that content may more or less resemble the actual phenomenon represented as MMs always capture only a limited representation, ranging from similarity or analogy (being *like* the thing) to isomorphism (being *as* the thing; Johnson-Laird, 1983). Altogether, MMs are token-assemblages representing distinct possibilities for a situation (Johnson-Laird and Byrne, 2002) and the likelihood of a particular MM guiding one's interpretation of a situation depends on its accessibility. That is, a MM may be invoked when one of its components is made salient by some priming element of an immediate situation (see Roskos-Ewoldesen et al., 2004). For example, if a person consumes repeated media depictions of silver, humanoid robots then “silver” and “humanoid” may become deeply ingrained components of that person's MM for robots.

The question of robot MM *structure* is a matter of how those knowledge tokens are associated and how those associations give rise to the overall structure of the model. For both considerations, if we think of MMs as comprising individual nodes (tokens) that are linked together in a network (i.e., a connectionist approach; see Medler, 1998) then it is useful to draw from network principles to consider links among held ideas (see Woods, 1975). From this frame, individual tokens may serve as links between other parts of the MM (signaling conceptual or functional bridging) and may be variably central to the MM (signaling importance to the phenomenon). Associations may exist between more- or less-similar tokens (signaling copacetic or tensioned relations), may be directed or reciprocal (signaling influence balances or differentials), and may be singular or multiplex (signaling simple or complex relations). The MM structure, overall, may be more or less dense (signaling greater or lesser cohesion of the parts of the MM) and, relatedly, may feature structural gaps (signaling conspicuous lapses in understanding or opportunities for exploration). Because of these potential linkages among knowledge tokens within a MM, if a situation offers a cue that causes a person to access one part of that network, it may result in the “spreading activation” of other parts of that MM network (see Collins and Loftus, 1975, p. 407; Krcmar and Curtis, 2003). To extend the previous example: if the repeated media images of silver, humanoid robots also frequently depict them performing violent actions, then “violence” may also become deeply ingrained in the MM for robots. When the person holding that MM actually encounters a silver, humanoid robot (primes that activate those portions of the MM), the violence components of the MM may in turn be activated such that the person assumes that robot will be violent—even if it gives no indication of harming anyone.

Understanding MMs for robots as a social technology is of central importance to understanding this form of human-machine communication because humans make decisions about communicative action based on their conscious or preconscious cognitions of social others—no matter whether these social others are human or non-human (Nass and Yen, 2010). For instance, one may be more or less likely to engage another person based on self-sameness or difference (Liviatan et al., 2008), stereotypes and norms (Bargh, 2006), or more general patterns of relational facts (Fox, 1967), and many such human-human relational dynamics are mirrored in human-machine relations (Nass et al., 1994; Spence et al., 2014; Bowman and Banks, 2019). However, social robots also deviate from humans in appearance (Duffy, 2003), signals of intelligence and agency (Ullman et al., 2014), and ability to adapt to novel communication scenarios (Salem et al., 2015) so it is necessary to take a step back and consider the nature and structure of held MMs for robots. This is especially important because MMs for robots may be invoked in a range of applied communication scenarios, engendering implications for interaction design to varyous ends. For instance, social robots may serve learning or socialization goals that do not rely on a profound technological understanding of their mental architecture—such knowledge even might be detrimental to their purpose. Alternately, it may be important for humans to have a clear and accurate MM of robots as technical systems, especially in high-stakes collaboration contexts (Phillips et al., 2011).

Extant research has focused on *inferences* of people's MM content based on their estimations of a robot's knowledge (Lee et al., 2005), social or visual ratings (Powers and Kiesler, 2006), quantified trait evaluations (Kiesler and Goetz, 2002) or on *assumptions* about MM content used to manipulate interaction experiences (Kwon et al., 2016). One study focused on children's understanding of robots as having animistic cognitive, affective, and behavioral characteristics, however the questions eliciting that understanding were specific to a single co-present robot (Beran et al., 2011). Thus, in considering the nature of MMs for robots, it is first necessary to *inductively discover* the nature of human-held MMs for robots, broadly. To that end, this investigation first asks: **(RQ1)** What is the (a) content and (b) structure of people's mental models for actual social robots?

### Media Cultivation of Robot Mental Models and Social Judgments

In 1976, in the course of laying out Cultivation Theory, Gerbner and Gross argued that television as a “new symbolic environment” was likely to be “the chief source of repetitive and ritualized symbol systems cultivating the common consciousness of the most far-flung and heterogenous mass publics in history” (p. 174). More than 40 years later, print and television are joined by myriad digital technologies in contributing to the shared and disparate understandings of an arguably even more heterogenous public. Although the framework originally attended to television, print and electronic media also help shape social realities through consumption volumes, convenience, accessibility, and (to some degree) narrative. These dynamics, as espoused in that theoretical framework, may all apply to both older and new forms of media (Morgan et al., 2015) despite increasing content and format heterogeneity.

The ways that media shape social realities can perhaps be best understood in terms of their contributions to humans' MMs for the world. Roskos-Ewoldesen et al. (2004) argue that specific media representations (depicted events comprising time, space, causality, intentionality, and agents; Zwann, 1999) are mentally indexed during media consumption, giving rise to cognitive representations of situations that—with greater cognitive accessibility and greater alignment with extant MMs—guide how we interpret events in our immediate world. From this frame, MMs are enduring but dynamic cognitive structures (Roskos-Ewoldesen et al., 2004) that intercede the direct consumption of media representations and the application of those representations to everyday life experiences. For instance, if a person with no immediate experience with artificial intelligence (AI) has consumed repeated representations of AI as digitally embodied and variably distributed (as is the case with Cortana in the videogame *Halo*, the Puppet Master in the manga *Ghost in the Shell*, and Wintermute in the cyberpunk classic *Neuromancer*), those properties are likely core to that person's MM for AI; upon encountering an actual, physically embodied robot they may have difficulties recognizing that robot as artificially intelligent in any way. MMs for discrete characters and character tropes materially contribute to knowledge structures, including lay theories about social agents and interactions (Schneider, 2001) as people make source-agnostic, heuristic evaluations during interactions (Shrum, 1998). If robots MM components are formed and reinforced by repeated exposures to similar (or similarly experienced) media representations, they may become chronically accessible from memory; those highly accessible concepts may become even *more* accessible for a short period of time when primed by cues in immediate exposures to an actual robot (see Roskos-Ewoldsen et al., 2009).

These social-evaluation individual effects and cultural shifts have been explored in cultivation research in relation to varied social judgments across varied populations (see Morgan and Shanahan, 2010 and for a review). For instance, heavier media consumption has been linked to more traditional gender-role attitudes among Japanese viewers (Saito, 2007), more unfavorable evaluations of Latino criminality in line with higher represented Latino criminality (Mastro et al., 2007), and differential rationales for socioeconomic differences among Black and White communities (Buselle and Crandall, 2002). Given (a) observed links between increased consumption of media and negative evaluations of non-majority populations, in line with disproportionately negative representations of those populations and (b) frequent popular-media representations of robots in popular culture as foils for what it means to be properly human (as othered fascinations, slaves, and artificialities; Kakoudaki, 2014), it is prudent to consider how the accessibility of robotic media characters may be associated with how people hold cognitive representations for actual robots as they exist in the world. Following, this study queries: **(RQ2)** What is the relationship between (a) recall and (b) evaluation of robot characters and the content of people's mental models for actual robots?

Further, robots are often represented as agentic entities in popular media; such depictions present opportunities for social agency to be incorporated into MMs and, following, for those cognitive structures to influence social evaluations in anticipation of or during actual encounters with robots. Regarding potentials for media-character exposure to influence social judgments of robots, it could be that salience of characters from past exposures signals the accessibility of the construct of “robot” as represented across exposures (cf. Sundar et al., 2016, in line with Roskos-Ewoldesen et al., 2004). It could also be that mere exposure effects manifest, with more frequent exposures to robot characters beget more positive attitudes toward actual robots independent of any particular recollection (cf. Zajonc, 1980). Considering potentials for MM influence on social judgment, it may be that through indexed robot representations' perceived iconicity (the interpreted likeness of a representation of a thing and the thing itself; see Alexander, 2010) functions as a cognitive framework or small-scale model of social reality (Craik, 1943) that grounds possibilities for a robot-as-agent and how it will behave. For instance, a MM with a central functional component (i.e., a robot is a weapon or a tool) could be a ground that reflects lower perceived social agency.

Indeed, past work has suggested that both manipulated narratives and ostensible MMs shape and order observations of and interactions with robots, especially in ambiguous encounters (Powers and Kiesler, 2006). Greater recollection of robot characters from film (particular humanlike robots for which sympathy was felt) was associated with lower anxiety toward robots (Sundar et al., 2016). More generally, narrative framing of robots may enhance perceived usefulness and adoption intention (Mara et al., 2013) and reduce uncanny responses (Mara and Appel, 2015) independent of robot morphology (Rosenthal-von der Pütten et al., 2017). Building on that work, the present study seeks to consider the potential for characters and MMs to directly influence interactions: **RQ3**: (How) are (a) character recall and (b) mental models for robots associated with social judgments of an actual robot?

## Methods

To address the posed research questions, an online survey was employed, inclusive of a partial replication of Sundar et al. (2016) design for elicitation of salient robot characters. An approximately nationally representative sample of U.S. residents (US Census Bureau, 2010) was recruited via Qualtrics sampling service. Among all participants (*N* = 410) were 50.5% female, 48.8% male,0.7% non-binary, and *M* = 46 (*SD* = 16.93) years old. They were 58.8% White/Caucasian, 15.1% Hispanic, 10.2% Black, 4.6% Asian, and 11.3% comprised other single or multiracial identifications. Highest education completed included: 1.5% less than high school, 12.9% high-school diploma or equivalent, 24.1% some college but no degree, 13.9% associate degree, 27.6% bachelor's degree, 20% graduate degree. Geographically, 34.9% live in the southern region of the United States, 18.3% in the northern, 22.4% in the midwestern, and 24.4% in the western. For annual household income, 17.1% made < $25,000, 23.4% earned $25,000-$50,000, 18.8% earned $50,000-$75,000, 13.2% $75,000-$100,000, and 27.5% earned $100,000 or more.

The survey took approximately 30 min to complete, and participants were paid by the sampling service for their participation. During initial data review, cases were removed when participants did not pass the audiovisual check, were speeding (having completed the survey in less than half standard deviation from the mean time), or were likely bots as signaled by unintelligible, illogical, or data-mined open-ended responses. Those removed cases were replaced with purposively sampled cases to ensure sample representativeness.

After completing informed consent documentation, the survey progressed through demographics capture for descriptives and quota sampling followed by an audiovisual access check to ensure participants could see and hear the video stimulus. Next, participants were asked to give open responses to questions eliciting their mental models for social robots. After that, participants were asked to (by memory only) name robot characters from different media forms (i.e., print, film/tv, and interactive media). Finally, participants were asked to view a video of an actual robot and then to respond to established scales for social impressions of that robot, as described below. This ordered procedure permits cautious inferencing of the ways that salient media characters (from past exposures) may implicitly influence unprimed explanations of what robots are that, in turn, may serve as a ground for judgments of actual robots. Complete instrumentation, data, and analysis documentation is available via online supplements: https://osf.io/4npgh/.

### Mental Model Elicitation and Analysis

Participants responded to three broad questions: What is a robot? What does a robot look like? and How does a robot work? Participants responded to the three questions with answers ranging from 3 to 430 words in length (*M* = 57.49, *SD* = 47.55). Cases were retained for analysis if participants answered at least one of the three questions. Because MMs are proverbial black boxes—the cognitive structures of interest exist only in people's minds and they are usually not comprehensively or accurately accessible or expressible—these questions functioned as MM elicitations. Specifically, in the production of verbal or written language, a person takes internal knowledge and externalizes it, translating the non-linear MM into a linear sentence that can be understood by another (Rickheit and Sichelschmidt, 1999). In this translation, words in the sentences represent knowledge tokens, and the grammar of the sentence signifies the relationships among the words—and so among the tokens (Johnson-Laird, 1981). Following, the structure of a MM may be inferred by eliciting descriptions of a phenomenon and identifying patterns in the words and relations among them (Sowa, 1992).

Because MMs rely on a wide range of knowledge sources and because each model can be vastly different from the next, they are inherently “messy, fuzzy … ill-defined, and essentially unbounded” and require a constraining the considered domain (Sanford and Moxey, 1999, p. 60). To constrain participant's considerations of robots, the questions' emphases on robots' nature, appearance, and function were selected because they reflect current understandings of individual differences in human-robot interactions as a function of visual, behavioral, and perceptual markers of ontological categorization (Kahn et al., 2011). These data were aggregated for each participant as representative of accessible portions of that person's MM, and then the response corpus for all participants was first analyzed in aggregate and then by individual respondent. This analysis was conducted using Leximancer: a semantic network analysis (SNA) tool that conducts semantic extraction (induction of concepts via constitutive keywords, the meanings of which are inferred through co-occurrence with other words) followed by relational extraction (coding of text segments as featuring the concept and subsequent count of concept frequencies' relative co-occurrence; Smith and Humphreys, 2006). In other words, it induces the meaning of specific words and higher-order concepts by their context and then calculates the probability of finding a second concept given the presence of the first. Leximancer is an appropriate tool for this analysis because it facilitates identification of both semantic and structural patterns in texts (here, in elicited MM-indicative texts).

### Robot Character Recall Validation

For character recall, participants were asked to name by memory as many robot characters and their source titles as possible, up to three characters each for print, film/tv, and interactive media (randomly ordered). Where the name or the title could not be recalled, participants were asked to provide a description or synopsis. Recalled robot characters were validated by ensuring that a character of that name or description was present in that title by comparison against Wikipedia articles, fan synopses, or the media text itself (in that order, progressing as necessary for confirmation). For descriptions or synopses, the information given was used to make a similar validation to the extent possible. When the character could not be validated, it was removed from analysis; the potential for false invalidation due to incomplete, vague, or insufficient descriptions is acknowledged as a limitation of this study. Through this process, it became clear that some participants named actual, manufactured robots (rather than fictional characters) that they had seen depicted in different media. Because answers indicated that most recalled actual robots were experientially associated with media representations (e.g., “the ones in the Verizon commercial”), all actual robots were retained as valid for inclusion in recall measures.

### Actual Robot Stimulus

Participants were shown a video of a “real robot named Ray” (RoboThespian by Engineered Arts, with the default model's rigid face and default male voice). The video is publicly available and produced by the robot's manufacturer (Engineered Arts, n.d.) and was edited by the researcher to remove branding information. Therein, the robot answers the question ‘Are you a real robot?' by describing the things it can do and asking viewers what they think as to whether or not he is real (see supplements for the complete video). A page timer prevented progressing in the survey until after the video had played completely.

### Measures

#### Mental Model Metrics

Each keyword was a node and co-occurrences among them were ties. The strength of the tie is the number of co-occurrences. Constellated keywords make up concepts, and constellated concepts make up the larger network. Statistics can then be calculated for the nodes (keywords/concepts), ties (co-occurrences), and the network structure as a whole. Specific measures are explained in relation to specific results in the next section.

#### Recalled Characters

The number of all valid robots was summed for each participants' total recall (*M* = 6.63, *SD* = 1.63, range 4–9). Medium-specific counts were: screen, *M* = 2.10, *SD* = 1.07 (commonly R2D2/C3PO, Terminator, WALL-E, Transformers); print, *M* = 0.61, *SD* = 0.91, (commonly Cyborg, C3PO); interactive, *M* = 0.42, *SD* = 0.83 (commonly Mega Man, R2D2, WALL-E, Terminator); actual, *M* = 0.36, *SD* = 0.80 (commonly Alexa, Roomba, Siri). Following Sundar et al. (2016) original design, each character was rated via single seven-point Likert-style items for its perceived badness/goodness and degree of felt sympathy for the character.

#### Social Evaluations of Actual Robot

Participant reactions to the actual robot video were captured using established scales. Mental capacity was measured using an early version (Malle, personal communication) of the Robot Mental Capacities Scale (Malle, 2019), with 20 items encompassing three dimensions: affective capacity (α = 0.96, *M* = 2.42, *SD* = 1.56), social-moral capacity (α = 0.94, *M* = 3.06, *SD* = 1.68), and reality interaction capacity (α = 0.70, *M* = 5.02, *SD* = 1.48). Moral capacity was measured using the Perceived Moral Agency Scale (Banks, 2018), with 10 items composing two dimensions: moral capacity (α = 0.91, *M* = 3.11, *SD* = 1.72) and programming dependency (α = 0.75, *M* = 5.81, *SD* = 1.38). Trust was measured using the Multi-Dimensional Measure of Trust (Ullman and Malle, 2018; Malle, 2019): reliability/capability trust (α = 0.88, *M* = 4.98, *SD* = 1.28) and ethical/sincerity trust (α = 0.92, *M* = 4.00, *SD* = 1.60). Willingness to engage with the actual robot was evaluated via a hypothetical invitation to collaborate with the robot on a project and a binary yes (accept) or no (decline) response. All items were otherwise measured via seven-point Likert-style scales.

## Results

To address the question of content and form of mental models for robots, patterns were first identified in the aggregate corpus of robot descriptions given that cultivated MMs are engaged here as a cumulative social phenomenon. Then, individual-level MM content was considered in relation to recalled robot characters/ratings and trust outcomes. This is an appropriate approach because it is highly unlikely that there can be some kind of “canonical form” among individual MMs that would permit clear comparisons across individuals (Woods, 1975, p. 16) such that using the aggregate model as a benchmark is more valid than comparing among individuals.

### Content and Structure of the Aggregate Model for Robot Knowledge (RQ1)

**Figure 1:**
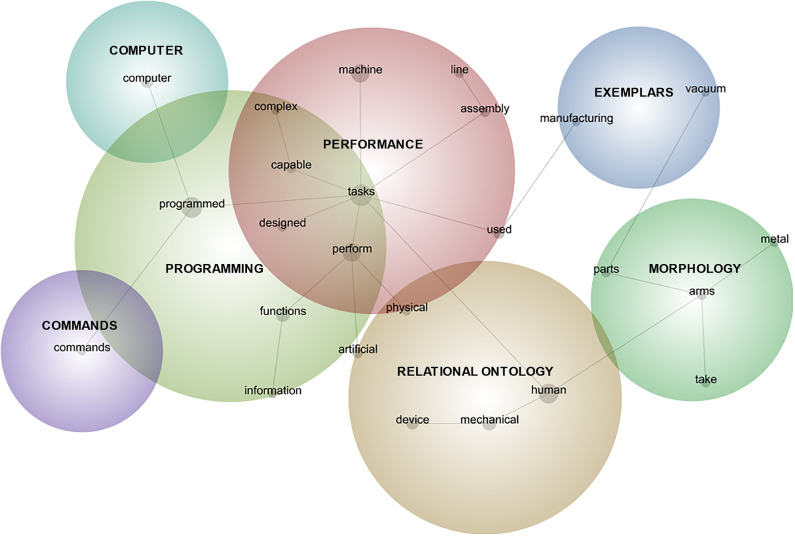
Aggregate semantic network map of robot nature, appearance, and function descriptions. Labeled network nodes are keywords identified by analysis software; bubbles are concepts induced by the software and interpreted by the author. Circle sizes only indicate conceptual boundaries and are not indicative of prevalence.

The semantic network analysis procedure using topical cluster mapping (see supplements for a complete analysis narrative) resulted in the induction of 25 keywords present in at least 5% of the data units (two-sentence blocks) across the corpus. To determine the most appropriate network model for further analysis, degrees of model granularity were explored: 0% granularity treats all individual keywords as distinct concepts and 100% granularity treats all keywords as comprising a single concept. Through iterative consideration of data in 10% granularity increases (see supplements for all iterations), the 40% granularity model was selected as most interpretable in relation to the research question and relevant literatures (Figure 1). Interpretability is defined here as a balance of specificity (level of detail toward comprehensive address of the research question) and coherence (each concept's keywords coalesce elegantly around a central and easily discerned idea).

The six concepts (i.e., clusters of identified keywords) were interpreted by comparing the induced keywords and concepts against the specific word-use instances to consider the contextual meaning of each keyword in relation to the other keywords in the induced concept. The concept was then considered in relation to extant literature for theoretical context and naming. This process resulted in the collapsing of two concepts because one was a subset of another through exemplification: *Exemplars* of manufacturing robots and robotic vacuums were common illustrations of robots principally focused on exemplifying ideas about *Performance*. The resulting six broad concepts in mental model content (RQ1a) and their constitutive keywords (in parentheses) are:

1. *Performance* (*n* = 609 data units in the corpus, inclusive of the integrated exemplar concepts): design for, fulfillment of, or ability to execute tasks, especially those that are complex or repetitive (tasks, perform, machine, used, capable, assembly, complex, line, manufacturing, vacuum). Although some highlighted performance in relation to talking or interacting (in line with Breazeal et al., 2004), most performance-related language highlighted task performance in a vague sense characterized by autonomous and intelligent action, but nearly always at the behest of humans (in line with Bryson, 2010).
2. *Relational Ontology* (*n* = 517): the intersection of non-human status or traits and the mimicry of, displacement of, association with, creation/design by, or control of, or operation by humans (human [inclusive of person/people], mechanical, device, artificial, physical). This complex concept comprises the intersection of the kind of thing robots are in relation to humans—and what they are not—encompassing appearance, behaviors, relations, and influences. In some cases, the sentiment distinguished humans and robots, some likened them, and some expressed a tensioned combination of similarity and dissimilarity (see Kahn et al., 2011).
3. *Programming* (*n* = 431): the coded inputs, informational resources, operating framework, and functional/subjective potentials, often according to the aims or imaginings of human designers (programmed, functions, designed, information). The presence of this component is in line with definitions of robots as engineered machines (e.g., Lin et al., 2011)—that they are made by someone or something and the nature of this making has fundamental influence on what robots do and how they do it. Often in tandem with a program as the ground for robot functioning, participants frequently mentioned information as the input, resource, or output of that functioning; information-focused functions are preferred by some people as a way of designating robot-appropriate jobs (Takayama et al., 2008).
4. *Morphology* (*n* =192): consisting of single or multiple parts or materials comprising a variably anthropomorphic form (arms, parts, take [the form of], metal). This MM component often featured general reference to “parts” (moving, interconnected, electrical, mechanical) or to specific parts (wires, gears, motors, sensors) that are manipulated in use or interconnected in construction. These descriptions were sometimes offered in tandem with (though sometimes instead of) notes about their composition of metal or plastic; mentioned forms ranged from single arm to an anthropomorphic or zoomorphic body, as well as to function-related morphologies (Yanco and Drury, 2004).
5. *Computer* (*n* = 141): containing, controlled by, or working in tandem with computational technology (computer). Although some participants described robots as being computers, being like computers, or containing computers, most referenced computers as controllers of the robots—as something apart from but intimately linked to its functioning. This may be interpreted as a form of perceived bicameralism (cf. Jaynes, 1976) in which the mechanical (present in the *Morphology* concept above) is the situated doer-of-things while the computational is the thinker- or decider-of-things.
6. *Commands* (*n* = 40): directives received and followed, usually delivered by or on behalf of human programmers or users (commands). Further illustrative of the perceived (or perhaps desired) centrality of humans to robot behavior, participants described robots as operating in response to commands received from a human or an ostensibly human-designed computer. Response to directives was represented as mandatory (“on command”) or elective, as coming in varied modalities (voice, text, button-pushing), and as instigating task performance. The appearance of commands as robot MM components may suggest a valuation of (or at least a defaulting to) human control over robots (see Gunkel, 2012).

In the course of this analysis, concepts were induced using the weighted values of keywords (how often those words occur within concepts compared to how often they occur elsewhere) as a measure of the contribution of the word to the induced concept (see Leximancer, 2005). However, in considering the structure of this network model (RQ1b), the unweighted number of links among concepts (the number of times that words from each concept co-occur within a data unit) were used because that metric considers rote co-occurrence (as a language-structure characteristic) and not semantic content of participants' language. The unweighted co-occurrence network was analyzed using SocNetV v2.5 (an open-source network visualization tool; SocNet, 2019). The resulting network model was analyzed for node, tie, and overall network statistics. See supplements for graph visualization and complete analysis outputs.

Regarding the overall network of 25 nodes and 260 edges (linked a total of 2,870 times in the corpus), the global clustering coefficient is the average of each node's proportion of actual links compared to possible links, indicating how tightly-knit the network is (0 is completely unlinked and 1 is a completely linked network). The measure is interpreted here as the extent to which the aggregate model is a collection of cohering ideas overall (a higher value, such that it can be said to be a model of some*thing*) vs. representing disparate and only loosely connected ideas (a lower value, such that the model is only a representation of multiple loosely collected things). The global clustering coefficient was 0.899 indicating a fairly tightly cohering keyword network since it approaches a value of 1.

For individual keywords, node degree (the number of links it has to other nodes) ranged from 1 to 699. Additionally, node centrality statistics indicate how powerful nodes are within the aggregate model. Here, these measures are used to discern how impactful a keyword (representing a component of a MM) is in relation to other keywords. Degree centrality is the simple sum of ties a node has with other nodes, interpreted here as the tendency for certain MM components to be more or less co-activated with other components. The components *tasks, programmed, human, perform*, and *machine* have the greatest degree centrality (Table 1). Betweenness centrality is the tendency of a node to sit on the shortest path between two other nodes, interpreted here as the extent to which a keyword may be co-activated with other pairs of keywords and linking them together in a MM. Only a few keywords had any measure of betweenness centrality: *tasks, human, programmed, machine*, and *performed*, suggesting those are the concepts that tend to bind together other concepts in participant language. Eigenvector centrality is a node's degree centrality proportional to the degree centrality of neighboring nodes, here signaling that a MM component is frequently co-activated with other components and/or is linked to keywords that frequently co-activate with other components. In other words, a high EC value (range 0–1) suggests a keyword represents a directly *and/or* indirectly influential MM component. In the robot MM network, *physical, taking* (form of), *manufacturing, designed*, and *device* had the highest values, suggesting that those components—though not as frequently mentioned—are most often linked to the network's most central keywords. Indeed, a review of data units reveals that in relation to their most common keyword link, each of those often serves as a modifier or modified word: *physical* performance, *taking* human form, *manufacturing* tasks, *designed* to complete tasks, and a mechanical *device*.

**Table 1:** Semantic network node property statistics for aggregate corpus of descriptions for robot category, appearance, and function.

**Keyword**	***n***	**Degree**	**DC**	**DC'**	**BC**	**BC'**	**EC**	**EC”**	**Most Common Link**
tasks	266	**699**	699	**0.122**	1,946.68	**7.053**	0.064	0.014	Perform (121)
programmed	326	**661**	661	**0.115**	1,045.52	**3.778**	0.070	0.015	Task (118)
human	344	**607**	607	**0.106**	1,103.30	**3.997**	0.067	0.014	Machine (88)
perform	170	**546**	546	**0.095**	165.10	**0.598**	0.096	0.021	Task (121)
machine	260	**533**	533	**0.093**	368.67	**1.336**	0.074	0.016	Human (88)
mechanical	143	333	333	0.058	0.00	0.020	0.108	0.023	Human (52)
functions	124	316	316	0.055	0.00	0.00	0.155	0.033	Programmed (58)
computer	141	251	251	0.044	0.00	0.00	0.216	0.047	Programmed (56)
device	87	230	230	0.040	0.00	0.00	0.249	**0.054**	Mechanical (41)
used	82	191	191	0.033	0.00	0.00	0.169	0.036	Programmed (20)
arms	66	141	141	0.025	0.00	0.00	0.203	0.044	Human (26)
artificial	62	140	140	0.024	0.00	0.00	0.190	0.041	Human (22)
designed	50	139	139	0.024	0.00	0.00	0.275	**0.059**	Task (28)
capable	41	121	121	0.021	0.00	0.00	0.182	0.039	Task/perform/machine (16)
assembly	35	111	111	0.019	0.00	0.00	0.207	0.045	Line (25)
complex	37	106	106	0.019	0.00	0.00	0.225	0.048	Machine (14)
line	27	88	88	0.015	0.00	0.00	0.188	0.040	Assembly (25)
parts	27	87	87	0.015	0.00	0.00	0.241	0.052	Programmed (11)
take	41	79	79	0.014	0.00	0.00	0.307	**0.067**	Human (13)
commands	40	67	67	0.012	0.00	0.00	0.148	0.032	Programmed (17)
physical	24	67	67	0.012	0.00	0.00	0.326	**0.070**	Perform (8)
manufacturing	25	63	63	0.011	0.00	0.00	0.288	**0.062**	Tasks (8)
metal	75	61	61	0.011	0.00	0.00	0.185	0.040	Human (14)
vacuum	32	59	59	0.010	0.00	0.00	0.221	0.048	Machine (8)
information	21	44	44	0.008	0.00	0.00	0.196	0.042	Programmed (8)

*DC, degree centrality summed; DC'%, degree centrality standardized index [DC/(n-1)]; BC, betweenness centrality summed; BC', betweenness centrality standardized index [BC/((n-1)(n-2)/2)].; EC, eigenvector centrality; EC”, eigenvector centrality standardized index (EC/ΣEC). Top five standardized values for each property are presented in bold*.

Regarding individual links between node pairs, tie strength is the relative weight of the link between two nodes, evaluated here in terms of the frequency of two nodes' co-occurrence within data units. Across the 260 edges, tie strength ranged from 1 to 121 (unconnected nodes were not considered in analysis), *M* = 11.09, mode = 1. These values indicate that the majority of keywords co-occur infrequently, while a small number of core keywords are more frequently and more tightly bound together in participants' language.

Altogether, to address RQ1 for the aggregate model, these metrics suggest that MMs for robots are best characterized as constellating around a few core of components (*tasks, programmed, human, perform, machine*) that are both frequently accessed and connecting other components together in the model. The relative infrequency and lower connectedness of the 21 other components suggests that people's MMs likely vary a good deal from person to person outside of those core components.

### Robot Mental Model Content and Character Recall/Evaluation (RQ2)

To explore individual differences in how salient media characters may be associated with MMs for robots, the presence of the discrete concepts in participants' language-indicated MMs for robots was analyzed for associations with character recall (RQ2a) and with goodness and sympathy evaluations of recalled characters (RQ2b). To do so, each participant's language was dummy coded (0/1 = absence/presence) for each of the six induced MM concepts identified above. A response was coded as having the concept present when it contained any keyword (i.e., node in Figure 1) belonging to that concept, as identified in the automatic semantic network analysis.

In participants' MM induction language, 77.6% featured *Performance* components, 79% included *Relational Ontology*, 71.5% included *Programming*, 41.2% included *Morphology*, 29.3% included *Computer*, and 9.5% included *Commands*. Regressing the total number of robot characters recalled upon MM components presence, there was no association, *R*^2^ = 0.135, *F*(6, 42) = 1.092, *p* = 0.383 (see supplements for complete outputs). To next consider whether MM components may be associated with evaluations of robot characters—independent of the number recalled—binary logistic regressions (presence/absence of each MM concept upon character evaluations) were conducted. This approach was taken because goodness and sympathy ratings for named characters were correlated (*r* = 0.607, *p* < 0.001). There were no significant models linking mean character goodness and sympathy ratings to the likelihood of indicating specific MM components (Table 2).

**Table 2:** Binary logistic regression for robot character evaluations and presence of mental model components in mental-model induction language.

**Variable**	**B**	**S.E**	**Wald**	**df**	***p***	**Exp(B)**	**CI for Exp(B)**
**Performance component:** **χ**^2^**(2)** **=** **1.174**, ***p*** **=** **0.424, Nagelkerke** ***R**^**2**^* **=** **0.006, 77.7% correct classification**							
Goodness	−0.076	0.124	0.372	1	0.542	0.927	0.726/1.183
Sympathy	0.138	0.104	1.739	1	0.187	1.148	0.935/1.409
**Ontology component:** **χ**^2^**(4)** **=** **2.928**, ***p*** **=** **0.231, Nagelkerke** ***R**^**2**^* **=** **0.011, 78.9% correct classification**							
Goodness	−0.046	0.142	0.102	1	0.749	0.956	0.723/1.263
Sympathy	−0.132	0.122	1.188	1	0.276	0.876	0.690/1.112
**Programming component:** **χ**^2^**(2)** **=** **0.161**, ***p*** **=** **0.923, Nagelkerke** ***R**^**2**^* **=** **0.001, 71.7% correct classification**							
Goodness	−0.028	0.121	0.054	1	0.806	0.972	0.767/1.232
Sympathy	−0.012	0.103	0.014	1	0.905	0.988	0.807/1.209
**Morphology component:** **χ**^2^**(2)** **=** **1.349**, ***p*** **=** **0.509, Nagelkerke** ***R**^**2**^* **=** **0.005, 58.6% correct classification**							
Goodness	0.108	0.110	0.960	1	0.327	1.114	0.898/1.381
Sympathy	−0.102	0.093	1.180	1	0.277	0.903	0.725/1.085
**Computer component:** **χ**^2^**(2)** **=** **4.130**, ***p*** **=** **0.127, Nagelkerke** ***R**^**2**^* **=** **0.015, 71.0% correct classification**							
Goodness	0.108	0.125	0.742	1	0.389	1.114	0.872/1.423
Sympathy	0.097	0.105	0.854	1	0.355	1.102	0.897/1.354
**Commands component:** **χ**^2^**(2)** **=** **0.711**, ***p*** **=** **0.701, Nagelkerke** ***R**^**2**^* **=** **0.004, 90.3% correct classification**							
Goodness	0.146	0.177	0.678	1	0.410	1.157	0.818/1.637
Sympathy	−0.093	0.142	0.422	1	0.516	0.912	0.689/1.205

### Recall and Mental Model Influence on Evaluations of in an Actual Robot (RQ3)

The final analysis attended to whether perceptions of perceived moral agency of, perceived mental capacities of, trust in, and willingness to engage with an actual robot may be predicted by robot character recall (RQ3a) and/or MM content (RQ3b). Because of a small to moderate correlations among the dependent variables (see supplements for correlation table) these associations were considered together via multivariate analyses.

Regressing the number of recalled robots upon the aforementioned social evaluations of the actual robot, the model was not significant, *R*^2^ = 0.083, *F*(8, 40) = 0.454, *p* = 0.880 (see supplements for complete regression table).

To explore a potential association between character evaluations and actual-robot evaluations (both sets of which exhibited correlations), a canonical correlation analysis was performed. The overall model fit was significant (Wilk's Λ = 0.915, *F*(16, 786) = 2.246, *p* = 0.003), explaining 77.5% of variance shared between variable sets. There were latent functions, only one of which significantly explained variance in the model ($R_{c}^{2}$ = 0.07, *p* = *0.0*03). Only structure coefficients ≥ |0.45| were interpreted (per Sherry and Henson, 2005). The canonical function indicated that (significantly, but weakly) the more perceptibly good *and* the more sympathetically recalled the robot characters, the more likely one is to perceive the actual robot as having affective mental capacity, to feel reliability/capability and ethical/sincerity trust, and to accept a collaboration invitation (Table 3).

**Table 3:** Canonical solution for character evaluations in relation to actual robot evaluations.

**Variables**	**Function 1**		
	**Coef**.	**r_**s**_**	**${\text{r}}_{\text{s}}^{2}$ (%)**
**Set 1: media character evaluations**			
Mean goodness	0.300	**0.779**	60.68
Mean sympathy	0.790	**0.971**	94.28
*$R_{\text{c}}^{2}$*			0.07
**Set 2: actual robot evaluations**			
Moral agency (PMA)	−0.044	0.384	14.75
Dependency (PMA)	0.259	0.293	8.59
Mental capacity-affective	0.649	**0.513**	26.32
Mental capacity-social/moral	−0.459	0.340	11.56
Mental capacity-reality interactive	−0.148	0.150	2.25
Trust-ethical/sincerity	0.456	**0.713**	50.84
Trust-reliability/capability	0.359	**0.662**	43.82
Invitation acceptance	0.387	**0.578**	33.41

*Coef., standardized canonical coefficients; rs, structure coefficient; = squared structure coefficient. Structure coefficients greater than |0.45| are in bold*.

To evaluate the potential influence of MM components on evaluations of an actual robot, presence of each of the MM components (among which there were only a few small correlations) were separately regressed upon the dependent actual-robot evaluation variables. Only one of the six models was statistically significant. The increased likelihood of a programming component in one's MM was positively associated with seeing the actual robot as being able to independently interact with its environment and negatively associated with perceptions of affective mind (Table 4).

**Table 4:** Regression results for evaluation of actual robot in relation to the presence of each possible mental model component.

**Variable**	**B**	**S.E**	**Wald**	**df**	***p***	**Exp(B)**
**Performance component:** **χ**^2^**(8)** **=** **12.991**, ***p*** **=** **0.112, Nagelkerke** ***R**^**2**^* **=** **0.067, 77.1% correct**						
Moral capacity	−0.009	0.127	0.005	1	0.946	0.991
Dependency	−0.046	0.116	0.154	1	0.695	0.955
Affective mind	−0.129	0.133	0.939	1	0.332	0.879
Social/moral mind	−0.113	0.166	0.464	1	0.496	0.893
Reality-interaction mind	0.218	0.112	3.816	1	0.051	1.243
Capacity/reliability trust	0.048	0.143	0.115	1	0.735	1.050
Invitation acceptance	−0.121	0.132	0.845	1	0.358	0.886
**Ontology component:** **χ**^2^**(8)** **=** **6.450**, ***p*** **=** **0.597, Nagelkerke** ***R**^**2**^* **=** **0.018, 79.0% correct**						
Moral capacity	−0.098	0.127	0.598	1	0.439	0.906
Dependency	−0.109	0.122	0.806	1	0.369	0.897
Affective mind	−0.162	0.136	1.419	1	0.234	0.850
Social/moral mind	0.073	0.166	0.192	1	0.661	1.075
Reality-interaction mind	0.017	0.112	0.024	1	0.876	1.018
Capacity/reliability trust	−0.023	0.141	0.026	1	0.871	0.977
Collaboration accept	0.044	0.129	0.116	1	0.733	1.045
**Programming component:** **χ**^2^**(8)** **=** **26.386**, ***p*** **=** **0.001, Nagelkerke** ***R**^**2**^* **=** **0.068, 71.5% correct**						
Moral capacity	−0.164	0.118	1.936	1	0.164	0.848
Dependency	0.008	0.107	0.006	1	0.938	1.008
**Affective mind**	–**0.254**	**0.130**	**3.852**	**1**	**0.050**	**0.775**
Social/moral mind	0.089	0.158	0.319	1	0.572	1.094
**Reality-interaction mind**	**0.205**	**0.102**	**4.062**	**1**	**0.044**	**1.228**
Capacity/reliability trust	0.027	0.128	0.043	1	0.836	1.027
Collaboration accept	0.109	0.122	0.801	1	0.371	1.115
**Morphology component:** **χ**^2^**(8)** **=** **5.217**, ***p*** **=** **0.734, Nagelkerke** ***R**^**2**^* **=** **0.011, 58.0% correct**						
Moral capacity	0.069	0.105	0.435	1	0.510	1.071
Dependency	−0.017	0.097	0.031	1	0.860	0.983
Affective mind	−0.139	0.115	1.462	1	0.227	0.870
Social/moral mind	0.037	0.134	0.075	1	0.784	1.037
Reality-interaction mind	0.005	0.091	0.003	1	0.958	1.005
Capacity/reliability trust	−0.114	0.114	1.010	1	0.315	0.892
Collaboration accept	0.007	0.104	0.005	1	0.946	1.007
**Computer component:** **χ**^2^**(8)** **=** **9.934**, ***p*** **=** **0.270, Nagelkerke** ***R**^**2**^* **=** **0.041, 70.5% correct**						
Moral capacity	−0.286	0.119	5.797	1	0.016	0.751
Dependency	0.174	0.111	2.464	1	0.117	1.191
Affective mind	−0.060	0.125	0.228	1	0.633	0.942
Social/moral mind	0.250	0.145	2.986	1	0.084	1.284
Reality-interaction mind	−0.062	0.099	0.392	1	0.531	0.940
Capacity/reliability trust	−0.045	0.124	0.131	1	0.717	0.956
Collaboration accept	0.144	0.111	1.666	1	0.197	1.155
**Commands component:** **χ**^2^**(8)** **=** **4.824**, ***p*** **=** **0.776, Nagelkerke** ***R**^**2**^* **=** **0.045, 90.5% correct**						
Moral capacity	0.312	0.163	3.648	1	0.056	1.366
Dependency	−0.089	0.166	0.290	1	0.590	0.914
Affective mind	−0.332	0.205	2.613	1	0.106	0.718
Social/moral mind	−0.226	0.219	1.067	1	0.302	0.797
Reality-interaction mind	−0.019	0.147	0.017	1	0.897	0.981
Capacity/reliability trust	0.107	0.192	0.312	1	0.576	1.113
Collaboration accept	0.043	0.167	0.067	1	0.796	1.044

*Significant associations are presented in bold*.

## Discussion

The present study's findings indicate that aggregate mental models for robots consisted of a few key concepts (*Performance, Relational Ontology, Programming, Morphology, Computer, and Command*) and a few core keywords (tasks, programming, humans, performance, machine); individual MMs tended to tap into a small number of these core components, beyond which there was a good deal of individual difference (RQ1). There were no links between recalled robot characters (in either quantity or evaluations) and MMs for actual robots (RQ2). There was no association between recall quantity and evaluations of actual robots. However, findings suggest that character evaluations, holistically, may prompt differential responses to actual robots: the more sympathy one has for perceptually good robot characters experienced recalled from media, the more likely one is to engage in future collaborations with an actual robot, to trust it, and to perceive it has having a mind (RQ3a). Evaluations of actual robots may also be influenced by MM components: holding the notion of “programming” in one's MM was positively associated with reality-interaction mind perception and negatively associated with affective mind perception (RQ3b).

### Mental Models for Robots: Narrow Plus Nuance

This study's findings indicate that—at the aggregate level—collective ideas about robots as a class of agent are relatively simple, relying on a small number of higher-order concepts. Individual MMs more or less take up these concepts, beyond which there appears to be a good deal of difference as people draw from more nuanced characterizations. Broadly these findings align with work suggesting that robots' discrete physicals features (Powers and Kiesler, 2006) and task-related expectations (Kiesler, 2005) are important MM components, here as embedded within the higher-order *Morphology* and *Performance* concepts. Additionally, extant suggestions of humanlikeness (Kiesler and Goetz, 2002) as core to MMs were supported within the higher-order consideration of robots' *Relational Ontology* as functional, social, and embodied relations with humans. Components of *Programming, Computer*, and *Command* align broadly with those supported by some veins of the explainable-AI movement in which functional accountability of AI and associated robots should be driven by transparency in those technologies' inner workings (Wachter et al., 2017)—acknowledging a distinction between knowing that robots have these components and understanding how those components actually work. It should be noted that the presence of these factors—and not others—in the present analysis is not to say that those factors do not exist in people's MMs; indeed, aggregate mental models are likely to vary by population. It is merely to say that, especially for models-in-application in the perception of a particular robot (where personality characteristics may be important; Kiesler and Goetz, 2002), they did not appear to exist in cultivated, prototypical, societal understandings of robot as an ontological class.

This coalescing around a small set of concepts for what robots are and how they exist in the world is notable in that, from a Cultivation Theory perspective, *culture* is characterized as the knowledge required to interpret and predict the activities of a community (Goodenough, 1957). That the core, cultural knowledge comprising the ostensible “cultural consensus” (Roskos-Ewoldesen et al., 2004, p. 358) for robots relies on a simple set of concepts (principally their task-performative capacities, how they compare with or function in relation to humans, and their reliance on programming) has implications for how collective understandings may foster views of social robots as tools, as similar to but distinct from humans, and as designed actors. From some positions, these are useful paradigms for understanding robots as they preserve the distinctions from and primacy of humans (cf. Bryson, 2010, 2018). From other positions, these understandings could be seen as problematic since technological advances may increasingly engender robot agencies and, as a result, a duty to consider the obligation to afford them rights as authentic persons (cf. Gunkel, 2018). In particular, it is notable that holding in one's MM a *Programming* component was linked to increased reality-interactive mind perception (i.e., that it can manifestly function in the world—a requirement for service) and reduced affective mind perception (i.e., that it can think and feel; Malle, 2019). Practically, when individuals believe an agent's traits are fixed (as by rote programming or inherent personality) those individuals more strongly rely on trait evaluations to predict behavior (Chiu et al., 1991). Considering robot interaction potentials only according to assumed programming, then, may result in a reduced propensity to account for emergent, adaptive, or unprogrammed behaviors. Ethically, that combination of perceived high capacity to function and low capacity to affectively experience the world has historically been grounds for denial of personhood, as attributions of *doing* and *feeling* are consequential for the assignment of moral rights and acknowledgment of meaningful action (Waytz et al., 2010). That potential for appropriate or problematic objectification should be further explored as people increasingly use MM for robots to order their experiences of actual robots (Powers and Kiesler, 2006).

Beyond those ethics of ontological-class understandings, variations beyond a few core MM components hold implications for how human-robot relations should be explored in future research. Given the narrow set of mainstreamed ideas manifested in robot MMs, it may be in the nuances of these cognitive frameworks that propensities to accept or reject robots in varied roles (intimate companion, friendly acquaintance, co-worker, servant) may emerge. This is implicated in the finding that both trust and willingness to engage the actual robot were *not* associated MM components; as MMs broadly are understood to drive much of human meaning-making and so behavior (Miller and Johnson-Laird, 1976), it could be argued that robot trust and acceptance *must* be associated with some MM components outside of the higher-order concepts identified in the aggregate model.

### Media Character Evaluations Engender Actual Robot Heuristics

An alternative explanation for the non-association between MM content and evaluations of the actual robot may come in the potential for media-character evaluations to engender certain heuristics for considerations of actual robots. The link between sympathy felt for good robot characters and trust in, perceived mental capacity of, and willingness to engage with the actual robot—*independent* of MM content—may be interpreted as a combination of representativeness heuristics (Tversky and Kahneman, 1974) and pareidolia (seeing ordered patterns [often human faces] in random or unrelated information; Liu et al., 2014). That is, consideration of the plights of robot characters in their respective narratives may serve as social simulations (Mar and Oatley, 2008) engendering sympathy; in the absence of direct exposure to robots, those characters are engaged as exemplars for all robots and so the sympathy for characters manifests as a sympathetic heuristic for actual robots. Said another way, media representations may foster non-conscious affective orientations toward robots that are independent of conscious understandings of them. This interpretation aligns with dual-process approaches to human-robot interaction (e.g., Lobato et al., 2013) in which people have an automatic and mindless reaction (Type 1 processing) to machine delivery of social signals followed by a delayed and conscious evaluation (Type 2 processing), as has been exhibited in robot mind perception (Banks, 2019) and suggested for various other forms of social cognition (Wiltshire et al., 2015). In short, it may be that people develop a *sense* of what a robot is that may or may not line up with their *knowledge* of what a robot is (Airenti, 2018), and that sense may be primed for high accessibility in evaluations of actual robots (see Roskos-Ewoldsen et al., 2009).

The present findings have mixed alignment to those from Sundar and colleagues (2016), the study on which the present investigation was based. In that study of older adults' recall and robot attitudes, higher character recall was associated with lower anxiety toward robots and greater felt sympathy toward recalled robots was related to more positive attitudes toward robots. In the present study, recall volume had no association with any actual-robot evaluation metric, but there was alignment in the link between character sympathy and positive evaluations of and intention toward the actual robot. That recall volume effects were not seen here could be a matter of sampling differences and media formats considered: the original study focused only among older adults and only on film characters, while this investigation considered an age-representative sample from the U.S. and considered multiple media types. It could be that age is associated with temporal trends in media representations of technology or that media format carries similar norms for representations of robots, or more recent general availability and visibility of machine agents such as voice agents and robot vacuums. For instance, in many videogames, robots function as combat companions (as with Rush in *Mega Man*, Claptrap in *Borderlands*, and Wheatley in *Portal 2*) while contemporary films often portray robots as independent protagonists or antagonists. Given potential for the realism present in many films and for CGI to foster greater accessibility of media-character exemplars (Busselle, 2001), these potential effects should be teased out in future investigations.

The alignment in findings regarding character sympathy and positive evaluations of robots hold implications for fostering potential acceptance or rejection of robots. Specifically, improving social integrations through trust and agency acknowledgment—as is important in human-machine teaming scenarios (de Visser et al., 2018)—may be facilitated through fictional representations that foster sympathetic responses. As primitive human capacities, sympathy toward another can be fostered in media through first-person narration, representation of a character's internal states, and the imbuing of humanlike traits (see Keen, 2006). Indeed, the characters most commonly assigned the highest sympathy ratings are those whose narratives often account moral, emotional, or otherwise personal journeys: Optimus Prime, WALL-E, R2D2, C3PO, Baymax, Sunny, Terminator, Robby, Johnny5, Data, Ultron, Bumblebee, Claptrap, and Iron Giant. The formulation of sympathetic orientations may foster rules-of-thumb for thinking about robots as a class of agents, which in turn may drive interpretations of their behavior (Banks, 2019).

### Limitations and Future Research

As an online survey, this study is subject to the usual suspects in empirical research limitations: self-report reliance on experience accessibility, potential demand effects, and non-response. There is also the possibility that participants may have looked up character names rather than engaging in pure recall, although the frequency with which participants did not name a full suite of nine characters suggests this is not the case. Further, although there is inferential time order, there is not manifest time-order that supports a definitive interpretation of the causal links between media consumption, MM content, and evaluations of robots. Because a single robot was used to evoke post-recall evaluations, generalizability of findings to other robotic agents is limited. Future research should seek to experimentally or observationally evaluate this study's proposed causal linkages and the applicability of findings to other robots.

Additionally, the design of this study limits the scope of claims that may be made regarding the influence of media characters. Participants were asked to recall them holistically and independent of particular events, since that is how we may reflect on or draw from media experiences. However, because MMs may evolve through the indexing of specific narrative events (cataloging temporal, spatial, casual, motivational, and agentic components; Roskos-Ewoldesen et al., 2004), future research should consider whether/how specific kinds of narrated actions and contexts may contribute to evolving robot MMs. For instance, it may be that what is most salient in holistic recall are sympathy-invoking events (explaining the influence of character sympathy in the present findings) or that popular discourse-consistent concepts are those that are most encoded and reinforced (perhaps explaining the lack of links between recalled characters with relatively complex personalities and fairly simplistic and mechanistic MM components).

Although the non-alignment between media character recall and MM content may tempt a rejection of Cultivation Theory premises in the case of robots, it is important to note that this study focuses on recall (i.e., salient and accessible characters) rather than on consumption volume. Although recall was an appropriate first step to evaluating potentials for cultivation effects, future research should investigate links between robot-representation consumption volume and variability in relation to MM components, as well as whether and how consumption and cognitive frameworks may evolve over time and relative to societal norms. Given the possibility that MMs for robots may emerge from media *and* from other sources (e.g., experience, conversation, advertising), future research should also explore intersections of media and other sources in relation to popular understandings of these social technologies.

## Conclusion

This study's findings suggest that mental models for robots constellate around a few core concepts, but these models have no association with recalled robot media characters; holding notions of “programming” in one's MM content may facilitate simplistic but (contemporarily) factually correct thinking about robots as an ontological agent class, while sympathetic orientations toward robot media characters may function as a heuristic for robots that leads to trust, mind perception, and collaboration intent. Findings are interpreted to suggest that characters engendering sympathetic responses—from Optimus Prime to WALL-E and R2D2 to Sunny—may be a vehicle for facilitating social integration of robots, while fostering understandings of robots as programmed systems may inhibit that integration via reduced consideration of them as affective agents. Media representations of robots likely contribute to people's internalized models for those technologies such that HRI scholarship and robot development should consider not only how cues and dynamics of live interactions may influence relational processes and effects, but also the influence of anteceding cognitive frameworks that people *bring into* those interactions. Those understandings and heuristics function as ground against which immediate interactions are considered.

## Data Availability Statement

The datasets generated and analyzed for this study can be found in this article online supplements: https://osf.io/4npgh/.

## Ethics Statement

The studies involving human participants were reviewed and approved by West Virginia University Institutional Review Board. Written informed consent for participation was not required for this study in accordance with the national legislation and the institutional requirements.

## Author Contributions

JB conducted the entirety of this work, including study design, data collection and analysis, and report writing.

## Conflict of Interest

The author declares that the research was conducted in the absence of any commercial or financial relationships that could be construed as a potential conflict of interest.
